# Orthodontic management of a patient with Schwartz-Jampel Syndrome

**DOI:** 10.12669/pjms.342.14804

**Published:** 2018

**Authors:** Naif A. Bindayel

**Affiliations:** Naif A. Bindayel, Associate Professor, Department of Pediatric Dentistry and Orthodontics, College of Dentistry, King Saud University, P.O. Box 60169, Riyadh 11545, Saudi Arabia

**Keywords:** Dental, Management, Orthodontic, Schwartz-Jampel Syndrome

## Abstract

The orthodontic diagnosis and comprehensive management of a 12.5-year-old Saudi girl, diagnosed with Schwartz-Jampel Syndrome (SJS) is presented. SJS is a rare autosomal recessive disorder that affects musculoskeletal structures of the body with clinical manifestations in the maxillofacial region. While few reports in the literature have discussed the dental aspects of the syndrome, this paper attempts to describe the full course of a two-phase comprehensive orthodontic treatment, and discuss its findings.

## INTRODUCTION

Schwartz-Jampel syndrome (SJS, also known as chondrodystrophic myotonia) was first reported in 1962 as a rare autosomal recessive disorder.^1^ The characteristics of the syndrome include generalized myotonia, skeletal abnormalities with joint contractures, and facial dismorphism.^2^ Typical clinical signs of SJS in the maxillofacial region include narrow palpebral fissures, blepharospasms, hypertelorism, low-set ears, mandibular micro/retrognathia, and narrow & high arched palate.^3^ Other orofacial findings include clefts of soft palate & posterior hard palate, with bilateral middle ear effusion.^4^ SJS patients are known to display signs of muscular hypertrophy, stiffness, and pursed lips.^5^

Few articles in literature have touched dental descriptive and management aspects of the syndrome, while almost none discussed topics related to orthodontic diagnosis and treatment planning. This case report aims to illustrate orthodontic assessment and comprehensive management of a case diagnosed with SJS.

## CASE REPORT

A 12.5-year-old Saudi girl, diagnosed with SJS presented to orthodontic clinic regarding upper and lower crowded arches. Extra-orally, the patient had convex profile, retrognathic maxilla and mandible, competent pursed tense lips, low-set ears, and pinched strong chin. Facial musculature was tense, and cheek tissues were bilaterally dense with limited mouth opening. Intra-orally, the case was in mixed dentition stage with bilateral Class II half-unit molar relationship (as a result of mesial migration of upper first molars), on a skeletal Class-I base. The case was complicated by constricted maxilla, high palatal vault, deep bite, server upper & lower crowding, and delayed dental development.

### Treatment plan

A two-phase treatment plan was formulated. First phase was aimed at facilitating the eruption of permanent dentition. The plan included the placement of upper Nance button, and lower Lingual Holding Arch (LHA) appliances, after extraction of lower left first primary molar, and all four second primary molars to allow for lower first premolars’ eruption, and expedite the dental development. At the same time, upper first premolars were extracted to allow upper canine eruption, while the lower first premolars were planned for extraction once erupted in place.

The second treatment phase included re-evaluation of permanent teeth eruption and anterio-poeterior skeletal relationship, to start orthopedic maxillary Rapid Palatal Expansion (RPE) followed by comprehensive upper and lower fixed appliance therapies.

### Treatment progress

**Fig.1:**
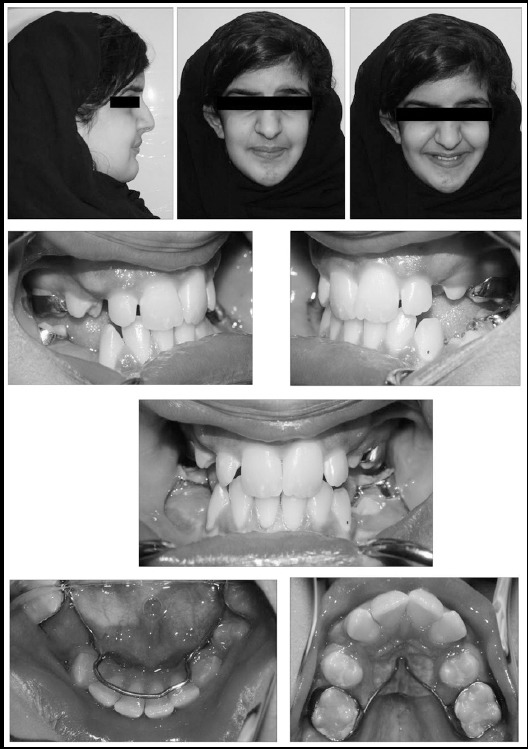
Pre-phase II orthodontic treatment extra and intra-oral photographs.

**Fig.2:**
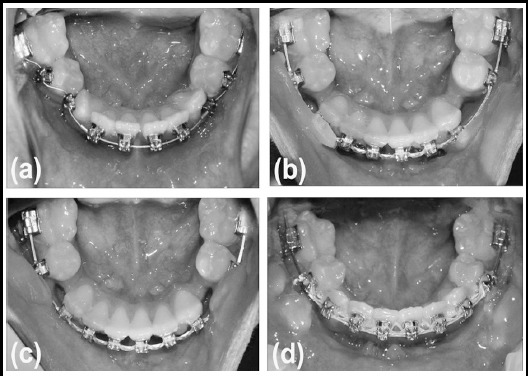
Occlusal view photos of the lower arch; immediately after bonding (a), with the application of Orthodontic wax (b), during the movement stage (c), and after spaced closed (d). Note the granulation tissues formed bilaterally on the buccal vestibule.

All planned extractions of primary teeth and upper first premolars were completed. Upon completion of phase I (Fig.1), the case was ready for RPE to aid in transverse correction and gaining additional upper arch space using Hass-type RPE. Patient was asked to turn the expander screw twice daily for the first three days, followed by once daily thereafter for one week. Upon follow up, central diastema (1 mm) was evident indicating a response of the mid-palatal suture. Despite the slow expansion progression, the case tolerated orthopedic transverse forces with no complication. Transpalatal arch was cemented to maintain transverse correction in the following months. After 11 months of Phase-I therapy initiation, the case was ready for fixed appliance treatment. The upper arch was bonded followed by bonding the lower arch after additional seven months. Following therapy initiation in lower arch, mucosal cheek laceration appeared resulting from the tense cheek tissues pressure on the thin round wire. This problem was managed by forming a soft bed using orthodontic wax and place it continuously against the arch wire for protection. During the subsequent visits in four weeks, the laceration healed and thick granulation fibrous tissue developed in response to the archwire trauma. Patient continued the use of orthodontic wax until the wound was healed (Fig.2). Treatment duration of the lower arch until space completely closed lasted for nine months, with total duration of two years and seven months

An upper and lower removable clear retainer was provided (for bed-time use) and the case was referred for periodontal care to manage the pre-existing lower thin labial plate of bone. The periodontist performed lower labial frenectomy and free gingival grafting under local anstehisa, after the orthodontic therapy was completed.

**Fig.3:**
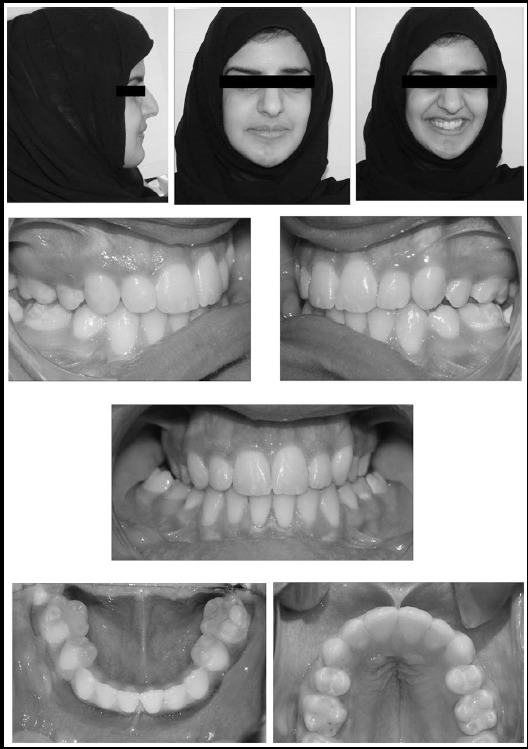
Four months post-phase II orthodontic treatment extra and intra-oral photographs.

Four months in retention phase (Fig.3), the case was stable, however, the patient complained from stiffness bilaterally in the cheeks upon waking up in the morning. Consideration was given to shift the retention protocol to Hawley retainer in case the symptoms persisted. The discomfort could be attributed to the abrupt increase in vertical dimension caused by wearing the clear retainers, and its impact of stretching the stiff facial musculature, mainly the masseter muscle. Nevertheless, the patient tolerated the retainer afterward and continued using the same appliance.

## DISCUSSION

The prevalence of SJS was reported to be low, with about a hundred cases reported since its first description in 1962.^3^,^6^ However, due to the common consanguineous marriages in Saudi Arabia, cases appear more frequently in medical literature. Five Saudi children, three males and two females, belonging to four families and displaying identical clinical features of SJS have been reported.^6^ The major orthodontic management challenge in cases with SJS is to manage the myotonic nature of facial muscles. The cheek tissues in the present case were affected bilaterally due to its tight adherence to dentition. The literature evidently addressed alteration of facial musculature in patients with SIS.^7^ Electromyographic analysis of facial muscles of two siblings showed an overall increased muscular activity of the temporal, orbicularis oris, orbicularis oculi, and masseter muscles.^8^ Thus, teeth crowding in the present case could be referred partly to the strong perioral musculature contraction.

Few reports have discussed oral and dental findings in patients with SJS. Dental findings were described in two siblings presenting with rigidity of Temporomandibular Joints (TMJ), accentuated contraction of the perioral muscles, limited mouth opening (30 to 35mm), and delayed bone development by approximately two years.^9^ Furthermore, SJS was identified as a predisposing factor for development of dentigerous cysts; advocating its surgical removal to avoid future complications.^10^

A key factor in SJS dental management is to minimize administration of general anesthesia to avoid development of malignant hyperthermia, one of the commonly associated complication with this syndrome.^2^ The utilization of customized devices to manage SJS cases was also emphasized, along with adapting specific techniques for each patient.^11^ In the current case orthodontic wax continuously used to protect buccal mucosal tissues (Fig.2). Treatment duration of phase-two with fixed appliance was two years and a half, although the remaining spaces requiring closure were limited, after alignment completion. The cranio-mandibular bone and muscular structural alterations in patients with SJS could be the reason behind this witnessed slow boney remodeling process in response to the teeth movement.

In summation, patients with SJS often present with crowded teeth in relation to altered musculoskeletal structures. A key role in orthodontic care of the present case involved early management and planning to facilitate eruption of permanent dentition. Consideration must be given to tense circumoral tissues and slow teeth movement while planning orthodontic management.
